# New Remedies and Old Diseases

**Published:** 1912-05-18

**Authors:** 


### The Hospital

The Workers' Newspaper of Administrative Medicine and Institutional Life, Administration, National Insurance and Health.

No. 1349, Vol. LII. SATURDAY, MAY 18, 1912. PRICE ONE PENNY.

### NEW REMEDIES AND OLD DISEASES.

The fact that the subject of consumption and its tuberculin treatment is now much under notice gives special interest to the lately issued annual report of the King Edward VII. Sanatorium. In the last few pages Dr. Radcliffe gives his laboratory statement, and this contains an interesting account of a tour round many Continental institutes for research, and of the work on tuberculosis there witnessed. In Koch's laboratory the effect of digestion on tuberculin, and the connected subject, the relative advantages of its oral and hypodermic administration, seem to have been investigated something like adequately. As a beginning old tuberculin was exposed for several hours to the action (*a*) of natural, (*b*) of artificial gastric juice. The digested product was then tested: (1) On patients by von Pirquet's method, when it was found to have lost much of its specific potency; (2) by intra-peritoneal inoculations on tuberculous guinea-pigs, when the digested tuberculin did not cause any reaction, whilst a similar injection of pure tuberculin caused the death of the guinea-pig, with symptoms characteristic of tuberculin poisoning; (3) by the complement fixation test: a considerable reduction in specific body content is stated to have been the result, and a still further one if trypsin digestion were superadded to the action of the pepsin. Further, enormous doses of old tuberculin by the mouth caused no general or focal reaction; conferred no immunity to a small dose given hypodermically, or to von Pirquet's reaction; and never caused a former ophthalmic or cutaneous reaction to flare up again, although this is usual when the hypodermic method is used. It may well be presumed that, as Dr. Radcliffe is not afraid to assert, the oral administration of tuberculin is futile.

Now many will remember it was claimed not long ago that tuberculin *per os* was just as effective as tuberculin *sub cutem*, the evidence adduced being that of general clinical observation and of the subsequent behaviour of the opsonic index. With regard to the latter ground, one may say without disrespect to the great inductive talent of Sir Almroth Wright, that most actual workers are agreed as to the chameleon-like behaviour in general of the tuberculo-opsonic index in phthisis pulmonalis. Cases there may be that show regular negative and positive phases in relation to exercise (as first shown by Wright's pupil Meakin, and Wheeler) and to inoculation, or which reveal distinct correlation of the opsonic index with the temperature; but there are plenty which do not. Moreover, the possibilities of research in this direction are sharply limited by time, since the determination of the index is a lengthy business, and for results to be strictly comparable the estimations taken successively at various hours of the day should all be done by one man. There remains then the other reason, the impressions left by clinical observation. As to this ground of opinion, it is surely time something were said.

Just at present there are at least four reputable remedies extolled as certain cures, unaided, of early consumption. They are "dioradin," "pneumosan," Dr. Lees' continuous antiseptic inhalations, and Dr. Camac Wilkinson's tuberculin course. Little but good is now heard of them all. We do not say of any one that it will turn out fallacious, but the reflection that all four can hardly collectively expect a better fate suggests the witty Frenchman's advice, to "be quick and take that new medicine while it still cures." The shortest glance at the history to date of phthisio-therapeutics unhappily confirms scepticism. In the stage of being evil spoken of are now Spengler's JK and Kuhn's suction mask. Obsolete quite are von Behring's tulase, Maragliano's serum, Moeller's timothy-grass bacilli, Barton Wright's succinimide of mercury, and many congeners of each. Then begins the vista of the original tuberculin, intravenous injection of antiseptics, cod-liver oil, hypophosphites, countless respirators, and so on, back to mediæval times and witches' broths. Up to now, and with all its faults, in the whole long history of the treatment of consumption the only landmark is the sanatorium.

But with very few exceptions each of these little separate efforts in the long search has been hailed as successful. That surely suggests that the chronic, often varying course of consumption is a most deceitful thing, and further, since the same thing is seen in respect of other diseases, that as a profession we have—most of us—an incomplete sense of the complexity of nature and of that great category, cause and effect. The cure for this failing must obviously be in the nature of a penalty, say distinct loss of prestige when the acumen of anyone—be he received authority or not—has clearly been shown at fault. At present, in the sphere of therapeutics, we make unsupportable statements rather recklessly and suffer little mortification when they get exploded. "Never be ashamed," said a famous lay teacher of the last generation. It is a vital trait enough, a trait of the successful in all but the highest walks of life, but hardly radically characteristic of the true leader of medical science.

Unfortunately, the unreflecting public, whose ever credulous gullibility seems to be fully as great among the educated classes as among the manual workers, unconsciously fosters and rewards this characteristic. He whose statements are always dogmatic and unblushing, whose capacity for self-deception is as overgrown as his conscience is atrophied, whose talent for self-advertisement has been developed by long practice: that is the man whom the public most esteem, and to him they will pay high fees ungrudgingly. And since the cause of this is to be found deep down in the foundations of human nature—the one thing of all others that changes least—it is idle to suppose that any system of education will change it. To bring up the young (medical) idea upon Bacon and Jevons and Darwin would be an excellent plan, were it not that the curriculum is already overloaded and that clinical studies are already overbalanced by the preliminary sciences. But even then the mammon worshipper will quickly see his opportunity and use it for accumulating wealth quickly, while the man who sticks to pure science is hard put to to make a living.

